# Propositional density: cognitive impairment and aging

**DOI:** 10.3389/fpsyg.2024.1434506

**Published:** 2024-08-29

**Authors:** Chaimae Harrag, Abdelkader Sabil, Manuel Célio Conceição, Gabriel A. Radvansky

**Affiliations:** ^1^Arts, Languages, and Literature, Faculty of Languages, Arts, and Human Sciences, Hassan 1 University, Settat, Morocco; ^2^Language Sciences, Faculty of Human Sciences, University of Algarve, Faro, Portugal; ^3^Department of Psychology, University of Notre Dame, Notre Dame, IN, United States

**Keywords:** propositional density, cognitive aging, cognitive impairment, dementia, aphasia

## Abstract

It is important to understand the relationship between cognitive abilities and language processing. Here, we explore a burgeoning area of research that harnesses semantic indices to predict cognitive impairment and track cognitive decline. One such index, *propositional density*, quantifies the information conveyed per language segment. Despite some variation stemming from methodological, sampling, and measurement differences, we suggest that propositional density has diagnostic and assessment value. This paper surveys existing studies that have used propositional density in the context of cognitive aging and impairment and offers some insights into the use of this index to highlight differences in cognition. We also suggest further explorations of basic research involving this concept, and some applications for assessing cognitive health.

## 1 Introduction

Exploring and understanding the interplay between language processing and cognitive abilities is a longstanding pursuit. Here we discuss how research has yielding insights into how cognitive processes shape, and are shaped by, language, with an emphasis on how the theoretical concept of propositional density can advance some research areas. We place a special emphasis on work on cognitive aging and cognitive impairment. Cognitive aging has been characterized by a decline in some mental abilities that are closely tied to language production (Craik and Salthouse, 2011; Mortensen et al., 2006) and comprehension (DeDe and Flax, 2016). Moreover, cognitive impairment, ranging from mild cognitive deficits to more severe neurodegenerative disorders, has an influence on language production (Bayles et al., 1993; Kempler and Van Lancker, 2002) and comprehension (Maseda et al., 2014). For instance, there is a decline in syntactic complexity of language produced by people with some cognitive impairment (Sand Aronsson et al., 2021). Also, impairment in some language processes is found in patients with Alzheimer Disease (Fraser et al., 2016). Our aim here is to overview work on propositional density as an index of linguistic complexity to show that it can then be used as an effective tool in research.

As detailed in the next section, *propositional density* quantifies the number of propositions (simple idea units) relative to words present (Brown et al., 2008). It has emerged as a diagnostic tool for studying cognitive aging (Kemper et al., 2001; Kemper and Sumner, 2001; Snowdon et al., 1996) and impairment (Snowdon et al., 1996, 2000) but has not gained the wide-spread use, even though it has the potential for assessing cognitive processes using both language production and comprehension. For language production, language samples can be assessed for their complexity and content. Changes in the density of these production samples can provide insights into current cognitive health, serve as early indicators of impairment, and help track any declines over time. This is based on the idea that cognitive declines would be revealed in simpler, less dense output. In contrast, for language comprehension people can be given language samples (e.g., sentences, paragraphs, or stories) that vary in their propositional density. Differences in understanding and memory for those samples can provide an index of cognitive performance (Stine and Hindman, 1994). While there is potential for using propositional density as an index of cognitive processing, there has been some variation in its application (Spencer et al., 2012). Here, we suggest that the major source of this variation centers on the use of different methods and populations, along with differences in scoring methods (Chand et al., 2012).

In this paper, we first provide a definition of propositional density. Then we overview some major findings and variations in the literature within the context of how propositional knowledge is represented and processed in memory in the domains of aging and cognitive impairment. Finally, we suggest some future directions of exploration, some of which inform our own research.

## 2 Defining propositional density

In philosophy and psychology, propositions are defined as fundamental units of meaning. They served as the basis for early models of human memory (e.g., Anderson, 1983; Anderson and Bower, 1974). In language, propositions are the smallest components assessable for their truth value, consisting of a predicate and an argument, each with unique semantic roles (Kintsch, 1998; Van Dijk and Kintsch, 1983). For example, in “John loves Mary,” the predicate is “loves” and the arguments are “John” and “Mary.” Sentences may contain multiple propositions, such as “John loves Mary deeply,” which includes “John loves Mary” and “John’s love is deep.”

Propositional density refers to the number of idea units within a linguistic segment, adjusted for the number of words. For example, “The old gray mare has a very large nose” contains five propositions: (1) the mare has a nose, (2) the mare is old, (3) the mare is gray, (4) the nose is large, and (5) the nose is very large. Propositional density is calculated as:

Propositional Density = Number of Propositions/Total Number of Words

For our example sentence, this would be:

Propositional Density = 5 propositions/9 total words = 0.56

It is important to note that propositional density is not simply a reflection of the number of words present. For instance, “The farmer in the field is growing wheat diligently, and he is wearing a blue hat” has a propositional density score of 0.375 (i.e., 6 propositions/16 words), while “The weary farmer, battling drought, planting wheat, seeking help, praying for rain, is wearing his hat” has a score of 0.563 (i.e., 9 propositions/16 words) despite both having the same word count.

It has been observed that propositional density scores are associated with differences in comprehension and memory. Increasing the number of propositions while holding word count constant leads to longer reading times (Kintsch and Keenan, 1973; Kintsch and Monk, 1972) and lower recall rates (Kintsch and Keenan, 1973). To wit, people with milder deficits would be expected to have difficulty with high density, but not low-density language. However, people with more severe deficits would be expected to have greater difficulty overall, and across a wider range of densities. Additionally, multiple propositions referring to a common concept increases retrieval time, as with the fan effect (Anderson, 1974). However, that said, memory is not solely guided by propositions. If people hear multiple sentences of overlapping content that refers to a common event, then they will use the degree to which memory probes match the integrated memory of all the propositions, rather than memory for the individual items that were heard (Bransford and Franks, 1971).

In the research discussed here, propositional density is used in two ways. First, it may be used to quantify the language produced by people. Specifically, how propositionally dense is their output? This is propositional density as a dependent variable. Second, it may be used to create different sets of materials, as was done by Kintsch, that vary in their densities. Specifically, this is done to assess language comprehension and memory. This is propositional density as an independent variable.

## 3 Cognitive aging

Cognitive aging brings about changes in mental function, such as slower processing speeds, diminished working memory capacity, and declining inhibitory function (Connelly et al., 1991; Park et al., 2002; Salthouse, 1990, 1996). These changes affect the ability to process and retain information, including some impact on language production and comprehension. For example, Kemper and Sumner (2001) found that older adults’ oral language samples were less syntactically complex, less propositionally dense, and shorter compared to younger adults. These changes were associated with processing efficiency. Less efficient processors tend to have lower propositional density scores and longer utterances. Moreover, lower working memory span scores were associated with decreased lexical variety and syntactic complexity. Here we consider age-related changes in cognition, and how these are related to changes in propositional density during language production, and how propositional density can influence language comprehension.

### 3.1 Language production

In terms of language production, as noted above, older adults are more likely to produce less propositionally dense utterances. Some research has suggested that changes in output density are a result of changes to cognitive processes operating prior to language formulation, *per se* (Madden et al., 2019). For language production models, this phase is called conceptual preparation (Levelt, 1993, 2000). During this time people select and assemble ideas that are the basis of language production. This requires executive control, involving decisions about the structure and content of the forthcoming message (Harley, 2016; Levelt, 1993, 2000).

Research indicates that age-related cognitive declines can impact conceptual preparation, and subsequently this is reflected in reduced verbal output and idea density (Kemper and Sumner, 2001; Mortensen et al., 2006; Soares et al., 2014). This conceptual difficulty is also revealed in a higher dysfluency rate for older adults when they are asked to talk about less familiar topics (Bortfeld et al., 2001; Mortensen et al., 2006). Because older adults have difficulty assembling the basic ideas that will underlie an utterance, they are more likely to have dysfluencies (um, er, uh, etc.) to give conceptual preparation more time to assemble the ideas that they are trying to convey.

More generally, word fluency reflects the ease with which people can generate multiple ideas and concepts. Such difficulties in idea generation then manifest themselves as declines in older adults’ speech (Clark et al., 2009). An exploratory analysis by Barker et al. (2022) suggested that executive functioning may be responsible for the inability to conceptually select between competing ideas and concepts. This then results in diminished propositional density. More specifically, underlying executive function deficits, such as decreased inhibitory abilities, may allow irrelevant concepts and associations to intrude, rendering the propositional language smaller and less coherent than that found with younger adults (Barker et al., 2020; Barker et al., 2022; Hoffman et al., 2018; Pushkar et al., 2000).

While changes in propositional density could reflect changes in idea selection (Barker et al., 2022; Frederiksen et al., 1990; Levelt, 1993, 2000), other work has suggested that such changes are associated with diminished abstract reasoning and executive function (Barker et al., 2022; Hoffman, 2018). Idea generation relies on executive resources (Alexander, 2006) to select relevant information so that any language output aligns with communicative intentions (Alexander, 2006). Thus, lower propositional density would reflect executive decline, such as is seen in older adults (Arbuckle et al., 2000). Moreover, it has been reported that declines in planning and producing language varying together result in a dual task cost (Kemper et al., 2001, 2009, 2011).

Overall, there is an age-related change in language production, with older adults producing less dense utterances. This may reflect challenges with underlying cognitive processes associated with the creation and assembly of the ideas underlying the language output. This is crucial for real-world tasks like eyewitness reports, where older adults may need adjustments to express memories appropriately, such as speaking slower, and taking more time to put their thoughts together.

### 3.2 Language comprehension

Age-related changes in language processing are due, in part, to changes in working memory (Gilchrist et al., 2008; Salis, 2011; Wingfield and Stine-Morrow, 2000). Specifically, older adults typically do not maintain as much information as younger adults do, and so are more likely to have difficulty processing more complex language and, thus, are more likely to show a deficit. Although some problems may arise from more syntactically complicated language structures (Norman et al., 1991), another significant factor could be how these age-related changes could give rise to difficulties in language comprehension with increased propositional density.

As noted earlier, research with younger adults has shown that increases in propositional density leads to longer reading times and decreased memory. The changes that accompany the natural aging process leading to greater challenges for older adults for denser texts. Essentially, declines in working memory capacity make it harder to manage more propositions in a text. This was found in studies by Fraser et al. (2016), Kemper et al. (2001), and Mitzner and Kemper (2003). As a result of older adults’ increased sensitivity to propositional density, they often need more time and resources to process denser sentences.

The impact of propositional density on comprehension for older adults was also seen in a study by Stine and Hindman (1994). This study compared reading and memory for younger and older adults for sentences with varying densities. Relative to younger adults, older adults spent more time reading denser sentences, and that this difference was correlated with working memory span scores. This was also associated with reduced memory. Stine and Hindman suggested that the slower reading time reflects a compensatory strategy with older adults taking more time to process the information because they are dissecting denser sentences into smaller, more manageable units. These smaller units could then be more easily handled with reduced working memory resources, allowing cognitive processes to be completed in a timely manner.

Overall, cognitive changes in older adults bring about changes in information processing. Materials that take into account these changes can enhance language comprehension and processing, supporting effective communication and lifelong learning. In essence, older adults may show a larger benefit to comprehension if presented with less dense materials.

## 4 Cognitive impairment

In this section, we consider two types of cognitive impairment, and how propositional density can be used as an index of the degree of disruption. These are the cognitive impairments that come with forms of dementia, such as Alzheimer’s Disease, and language specific deficits, such as aphasia.

### 4.1 Dementia

With dementia, subtle linguistic changes may signal early cognitive dysfunction, because language processing involves various cognitive mechanisms that are compromised (Caplan, 1993). Language production issues would reflect the downstream problems that result from such deficits. As an illustration of the usefulness of propositional density for assessing such cognitive changes, a study by Medina et al. (2011) examined the relationship among familial Alzheimer’s disease (FAD) mutation status, apolipoprotein E (APOE) genotype, and propositional density for non-demented people at risk for FAD. People provided biographical essays, which were analyzed for propositional density. Their results revealed no significant association between FAD mutation status and propositional density. However, the presence of the APOE Ε4 allele was strongly correlated with lower density.

Also, Engelman et al. (2010) found that cognitively intact people produce propositionally denser output than Alzheimer’s patients, likely due to their intact cognitive resources (Stine and Hindman, 1994; Stine-Morrow et al., 2006). In contrast, impaired people have limitations that hinder the production of denser output (Snowdon et al., 1996, 2000). More specifically, the propositional density of the output of language production tasks can be used to predict Alzheimer’s disease. As an example, Snowdon et al. (1996) analyzed autobiographies written by women around the age of 22 and compared them to subsequent outcomes. They found that lower density was associated with lower cognitive test scores and higher occurrence of Alzheimer’s disease later in life. Engelman et al. (2010) did a similar analysis using medical school admissions essays. Again, finding that lower density in the earlier writings of people who later developed Alzheimer’s disease. Thus, early life language production can be used as a predictor of later cognitive health.

A longitudinal analysis of language samples from healthy and adults with dementia showed a progressive decline in grammatical complexity and propositional density with age (Kemper et al., 2001). Alzheimer disease was accompanied by accelerated deterioration. Moreover, grammatical complexity decline was linked to digit span, while propositional density decline was associated with vocabulary differences. More recently, Mueller et al. (2016) assessed whether people with Mild Cognitive Impairment (pMCI) and memory decline exhibit deficits in connected language measures. The people described a picture, and these productions were analyzed for semantic content (total semantic units, propositional density, and unique words), syntactic complexity, and speech fluency. The pMCI group had fewer unique words and semantic units than the controls, and importantly, differed in propositional density. No differences were found in speech fluency tasks or syntactic complexity. Thus, measures of propositional density can capture cognitive changes that might be missed with other measures.

Propositional density can be used to detect declines in semantic memory, as is found with Alzheimer’s disease (Kirshner, 2012; Mascali et al., 2018; Zahn et al., 2004). During the early stages of the disease, people exhibit declines in semantic processing, as with lexical errors, delays in word finding, semantic paraphrases, and verbose language (Forbes-McKay et al., 2013), all of which reduce the density of their language productions. Venneri et al. (2016, 2018) suggested that tests of semantic processing, such as propositional density, can detect changes early on.

Supporting this, a study by Farias et al. (2012) examined whether density from oral language samples, obtained from cognitively intact, impaired, and demented groups, could predict subsequent trajectories of cognitive change. They found that density scores were more closely related to changes in overall cognitive function in the MCI and healthy groups than in the dementia group. This highlights the potential of density as a way to predict cognitive decline, particularly early on. Additionally, the study demonstrated that density was correlated with semantic memory, executive function, and spatial abilities, and minimally correlated with episodic memory.

It is important to note that all of the studies discussed in this section use measures of the density of language productions. To our knowledge, there are no studies of language comprehension and memory that use propositional density as a way of manipulating the materials presented and exploring language processing in this way. This is an open avenue for future research.

### 4.2 Aphasia

Propositional density has been used with nonfluent (Broca’s) aphasia patients to assess the informativeness and communicative adequacy of their language production (Barker et al., 2020; Bryant et al., 2013). In one study, Bryant et al. (2013) investigated the extent to which propositional density scores differed between aphasic and non-aphasic discourse, and whether it could adequately index the severity of the aphasia. The study included people from the Goals in Aphasia Project with post-stroke aphasia following a cerebrovascular accident in their language-dominant hemisphere, and their family members, who served as controls. The language production samples were analyzed for propositional density, lexical diversity, complexity (measured as the mean length of an utterance and number of utterances), and overall verbal productivity.

The results revealed that propositional density scores differed in the two groups. There was a negative correlation between these scores and the severity of aphasia, indicating that more severe aphasia compromised language production to a degree that results in less dense and information-impoverished discourse. These results were validated by correlations with other language measures including Number of Different Words (NDW), Mean Length of Utterance (MLU), and Number of Utterances (NU). An unexpected increase in the Type-Token Ratio (TTR) was observed in cases of aphasia, possibly due to the large sample sizes of aphasic language data in the present research.

Similarly, a study by Fromm et al. (2016) evaluated how proposition density can differentiate between people with aphasia and controls, as well as among subtypes of aphasia, based on procedural discourse and personal narratives. There were six aphasia types assessed: Broca’s, Wernicke’s, anomic, conduction, transcortical motor, and people that had an Aphasia Quotient greater than 93.8. The controls scored higher than people with aphasia on both tasks. Additionally, density scores differed among the aphasia types. Density scores for the Broca group were lower than those for all the others. Moreover, everyone with aphasia scored lower on discourse tasks than on the narrative tasks. This shows that propositional density can be used to distinguish between different types of language deficits and task types.

Some studies on aphasia indicate that even with reduced propositional output and impaired executive functioning, skills such as comprehension, repetition, reading, and naming often remain intact (Crescentini et al., 2008; Robinson et al., 2006; Robinson, 2013). Webster et al. (2018) found that propositional density did not impact reading time or accuracy in people with aphasia, indicating intact comprehension despite production deficits. Fromm et al. (2017) also observed no differences in comprehension between healthy people and people with aphasia. The deficits studied so far appear to be confined to language production. The intact skills along with reduced propositional language during discourse generation may not be strictly language-based but could also be attributed to an inability to select novel thoughts (Barker et al., 2022; Robinson et al., 1998, 2010). Difficulties in the sequencing and selecting of thoughts may hinder the fluid connection of ideas during language production. This may be why patients often do well on word and sentence-level generation tasks, which require generating only a single idea and focusing attention on the current message. Another possible explanation is that people with aphasia during comprehension use context and redundancy in connected speech, which enables them to infer meaning and compensate for their linguistic impairments (Huber, 1990).

## 5 Variation in propositional density

Propositional density has been useful for assessing linguistic ability and cognitive health, but there are challenges. These include variability in its reliability as a predictor of cognitive decline, effectiveness across modalities, study context, sample differences, and calculation methods. Each of these are considered in turn.

### 5.1 Predictability

While there have been studies showing that propositional density scores for language production can be used to help predict cognitive functioning in later life, there are also some inconsistencies. The predictability of propositional density scores from language output for later cognitive performance has relied heavily on rich production corpora. Ferguson et al. (2014) and Spencer et al. (2012) have both noted that longer writing samples tend to yield more consistent density measures, emphasizing the need for larger datasets to enhance reliability. Thus, it is expected that cases in which the language production output are smaller is likely to lead to less stable propositional density measures. Any predictions using such scores are likely to be less reliable.

### 5.2 Modalities

While there have been studies showing that propositional density scores for language production can be used as an index of cognitive functioning, there are also some inconsistencies depending on the modality of production. For example, Smolík et al. (2016) reported a decrease in density for amnestic mild cognitive impairment (aMCI) patients in spoken but not written language. This aligns with the Nun Study (Mitzner and Kemper, 2003), which found higher propositional density in written compared to oral samples. Written narratives tend to have higher density than spoken narratives. This difference may reflect the fact that the writing process serves to offload cognitive processes to some degree, which opens the door for denser language units.

### 5.3 Study context

As noted earlier, propositional density has been used in the context of both language production and language comprehension and memory studies. Each of these has its strengths and weaknesses. In terms of language production, it has clearly been shown that samples of early language production can, to some degree, predict cognitive deficits later on. This has not been demonstrated with language comprehension. That said, propositional density scores derived from language production require a large output sample to produce scores that are predictable. Moreover, in some cases, people may be less willing, or less able, to produce a great deal of linguistic output when asked, because, by its very nature, is cognitively demanding.

In comparison, for language comprehension tasks, researchers can use materials of various densities to assess performance. This can be done in a much shorter period and involves explicit experimental manipulation. This sort of assessment can involve both reading time measures, as well as memory measures. Such multimethod approaches are always preferred to single methods, such as just using memory. This also would allow for a better comparison between groups because the nature of the materials (the language input) would be the same in both cases. Any differences would be due to cognitive processes. That said, as noted earlier, comprehension measures are less likely to be useful in predicting later performance given the lack of background research on the topic, and the lack of test administration early in life. Still, overall, when faced with a choice, we would recommend going into any new evaluation to assess cognitive function using comprehension in situations where there is more task administration control.

### 5.4 Sample differences

Studies on conditions like MCI and aphasia report differences in propositional density due to the different cognitive profiles. For example, Mitzner and Kemper (2003) and Fraser et al. (2016) noted that Alzheimer’s disease impacts different brain regions, causing heterogeneity in language samples. Distinct subgroups, such as amnestic and dysexecutive deficit groups, may demonstrate varying effects on density.

In the context of healthy aging, Véliz et al. (2013) found that there are no differences in syntactic complexity and propositional density between healthy younger and older adults in sentence production. Similarly, Ferguson et al. (2014) noted that propositional density during production remained consistent in healthy women from young to mid-adulthood but began to decline in older adulthood. That said it should be noted that these studies involved small sample sizes (*N* = 20). Thus, it is important to consider the nature of the samples that are being worked with to best understand how propositional density scores reflect what sorts of differences in cognitive processing that are known to be present.

### 5.5 Genre influences

Propositional density scores can also vary with different linguistic genres (Alyahya et al., 2020; Fromm et al., 2016). For example, storytelling narratives often have more content words and lexical diversity compared to composite picture description and expository discourse. This may reflect the greater ease that narrative language is processed relative to other kinds. This ease of processing may free up resources, making it easier to integrate more basic idea units into a given segment of language.

### 5.6 Calculation methods

Several methods have been used to calculate propositional density. These include the Language Across the Lifespan (LAL) coding manual, the Computerized Propositional Idea Density Rater (CPIDR) tool, and Analysis of Idea Density (AID). Each of these are considered in turn.

LAL (Kemper, 1993) is an adaptation of Turner and Greene’s (1977) original manual defining propositional density. For this approach, propositions are grouped into three classes: predication (often verbs), modification (adjectives, adverbs), and connection (conjunctions, prepositions). The total number of propositions is divided by the total number of words.

The CPIDR tool (Brown et al., 2008) automates propositional density calculation by analyzing semantic content using parts of speech tagging, counting the derived propositions, and dividing them by the number of words. This is then followed by a refinement using post-analysis rules. These rules include flagging conjunctions, numerals, determiners, prepositions, adjectives, adverbs, possessives, verbs, relatives, or interrogatives as propositions. Additional rules condense complex verb phrases into single propositions; for instance, “may have been signing” would be condensed to a single proposition. Subject-auxiliary inversion is also used to correctly process questions. For example, “Has he come?” is converted to “he has come.”

The AID has been used for oral language samples Chand et al. (2012). It is based on Kintsch’s narrative analysis. This approach uses Turner and Greene’s (1977) classification (predication, modification, and connectives) but diverges by emphasizing the extraction of meaningful content, differentiating between semantic significance and grammatical structure. In this approach, an idea is only counted if it contributes to the overall meaning. This removes verbal asides that may occur during oral output (e.g., “Oh, hi there!”).

Thus, these guidelines also help distinguish between words and phrases that add new information versus those that fulfill grammatical necessities. For example, “And then, surprisingly, she left” counts “surprisingly” as it introduces a new idea, while “and then” is a narrative progression tool. Similarly, the repetition of “very” in “It was very, very cold” is evaluated based on whether it introduces new information or merely emphasizes the existing description. The manual also provides detailed guidelines on what constitutes a word, including lexical fillers, unfinished words, repeated words, utterance-initial conjunctions, and acronyms.

Overall, the LAL-based measure, CPIDR, and AID each have benefits and limitations. Incorporating propositional density into future research requires understanding the strengths and limitations of available tools. LAL provides a comprehensive analysis but is complex and less accessible without substantial linguistic training. CPIDR 3 segments text into propositional units using speech tags, offering an automated approach that simplifies the process but may overlook semantic richness and syntactic complexity. AID prioritizes semantic content, introducing rules to improve inter-rater reliability. However, it is labor-intensive and may allow for some element of subjectivity. This method relies on what the raters deem meaningful.

To illustrate differences among these measures, take the repetition of word very in “very, very cold.” In approaches such as LAL and AID, this may be counted as meaningful because it is used for emphasis, but another rater may discard it given it does not introduce new information. Thus, it is necessary to define what constitutes a “word” for accurate calculations. CPIDR 3 counts “very, very exhausting” as three words, while AID counts one “very.” Thus, clearer guidelines, such as standardizing the treatment of contractions, possessives, multi-word numbers, acronyms, and repeated words can ensure consistency in measurement, would be helpful.

## 6 Future research

Going beyond measurement issues, further work can be done looking at how this measure relates to various levels of language comprehension. There are three levels of representation that can be identified, namely the surface form, the textbase, and the mental model levels (Van Dijk and Kintsch, 1983). The surface form captures the verbatim wording of language, including the specific words and syntax that were used. The textbase captures the propositional idea units that are present in language, apart from the specific wording. For example, the sentences “The boy helped the girl” and “The girl was helped by the boy” have different wordings and syntax, but map onto the same underlying idea. Finally, the mental model level refers to what the language is about (Glenberg et al., 1987). It includes both information from the language itself, as well as inferences drawn from a person’s long-term knowledge. For example, if you read that the firecracker exploded, you are likely to infer that someone lit the fuse, even if it was not explicitly stated.

Propositional density primarily assesses the textbase level of language comprehension and memory, although there is some involvement of surface form knowledge as well, especially in the calculation of a propositional density score (i.e., the number of words used). However, it is unclear how this measure relates to the mental model level. This is important because research indicates older adults may perform similarly to younger adults at the mental model level despite differences at the textbase level (Radvansky and Dijkstra, 2007). Do propositional density differences also manifest themselves as higher level comprehension and memory differences as well?

Another issue is how propositional density affects memory over time, such as forgetting rates for denser language. Research has shown that different types of information follow different patterns of retention and forgetting (e.g., Fisher and Radvansky, 2018). For example, surface form information is forgotten quite quickly, within a few seconds or minutes (Sachs, 1967), whereas the mental model level may endure for days, weeks, or even decades (e.g., Doolen and Radvansky, 2021). It may be that language at different levels of propositional density may exhibit different patterns of forgetting. For example, it may be that denser texts result in longer lasting memory because there is more supportive informational content. Alternatively, it may be that denser texts actually result in shorter lasting memory because there is more information to encode, which taxes cognitive resources, resulting in poorer initial encoding.

Future studies should explore this and consider practical issues like eyewitness report accuracy, memory veracity, and confidence. Is propositionally denser output linked to greater accuracy, truth-telling, and confidence? This could be if a strong, more detailed memory trace is present, then there is more underlying information to draw upon. Therefore, this would allow language production to include more idea units within a given utterance.

Expanding propositional density as a tool to other cognitive domains offers promising avenues for research, particularly in understanding autobiographical memory and its relation to memory report generalization. For instance, changes in language production density could indicate the presence of conditions such as depression, which is often associated with over general memories that lack detail and specificity (Williams et al., 2007).

There is also a lack of studies in clinical settings of how materials of different propositional density affect comprehension in people with dementia, aging adults, and those with cognitive impairments. Most studies of these populations focus on language production. Understanding this could reveal valuable insights into the cognitive processes underlying language use, aiding in the development of better communication strategies and interventions. Extending this tool beyond its traditional applications could therefore provide additional insights into various conditions.

## Author contributions

CH: Conceptualization, Writing – original draft, Writing – review & editing. AS: Supervision, Validation, Writing – review & editing. MC: Supervision, Validation, Writing – review & editing. GR: Supervision, Validation, Writing – review & editing.

## References

[ref1] Alexander M. P. (2006). Impairments of procedures for implementing complex language are due to disruption of frontal attention processes. J. Int. Neuropsychol. Soc. 12, 236–247. doi: 10.1017/S1355617706060309 16573857

[ref2] Alyahya R. S. W. Halai A. D. Conroy P. Lambon M. A. (2020). A unified model of post-stroke language deficits including discourse production and their neural correlates. Brain 143, 1541–1554. doi: 10.1093/BRAIN/AWAA074 32330940 PMC7241958

[ref3] Anderson J. R. (1974). Retrieval of propositional information from long-term memory. Cogn. Psychol. 6, 451–474. doi: 10.1016/0010-0285(74)90021-8

[ref4] Anderson J. R. (1983). The architecture of cognition. Psychology Press: New York.

[ref5] Anderson J. R. Bower G. H. (1974). A propositional theory of recognition memory. Mem. Cogn. 2, 406–412. doi: 10.3758/BF03196896 21274765

[ref6] Arbuckle T. Y. Nohara-LeClair M. Pushkar D. (2000). Effect of off-target verbosity on communication efficiency in a referential communication task. Psychol. Aging 15, 65–77. doi: 10.1037/0882-7974.15.1.65 10755290

[ref7] Barker M. S. Ceslis A. Robinson G. A. (2022). Idea selection for propositional language production. Neuropsychol. Dev. Cogn. B Aging Neuropsychol. Cogn. 29, 260–283. doi: 10.1080/13825585.2020.1862044 33356844

[ref8] Barker M. S. Nelson N. L. Robinson G. A. (2020). Idea formulation for spoken language production: the Interface of cognition and language. J. Int. Neuropsychol. Soc. 26, 226–240. doi: 10.1017/S1355617719001097 31727185

[ref9] Bayles K. A. Tomoeda C. K. Trosset M. W. (1993). Alzheimer’S disease: effects on language. Dev. Neuropsychol. 9, 131–160. doi: 10.1080/87565649109540549

[ref10] Bortfeld H. Leon S. D. Bloom J. E. Schober M. F. Brennan S. E. (2001). Disfluency rates in conversation: effects of age, relationship, topic, role, and gender. Lang. Speech 44, 123–147. doi: 10.1177/00238309010440020101 11575901

[ref11] Bransford J. D. Franks J. J. (1971). The abstraction of linguistic ideas. Cogn. Psychol. 2, 331–350. doi: 10.1016/0010-0285(71)90019-3

[ref12] Brown C. Snodgrass T. Kemper S. J. Herman R. Covington M. A. (2008). Automatic measurement of propositional idea density from part-of-speech tagging. Behav. Res. Methods 40:540. doi: 10.3758/BRM.40.2.540 18522065 PMC2423207

[ref13] Bryant L. Spencer E. Ferguson A. Craig H. Colyvas K. Worrall L. (2013). Propositional idea density in aphasic discourse. Aphasiology 27, 992–1009. doi: 10.1080/02687038.2013.803514

[ref14] Caplan D. (1993). Toward a psycholinguistic approach to acquired neurogenic language disorders. Am. J. Speech Lang. Pathol. 2, 59–83. doi: 10.1044/1058-0360.0201.59

[ref15] Chand V. Baynes K. Bonnici L. M. Farias S. T. (2012). A rubric for extracting idea density from oral language samples. Curr. Protoc. Neurosci. Chapter 10:Unit10.5. doi: 10.1002/0471142301.NS1005S58 PMC3466486 23042498

[ref16] Clark L. J. Gatz M. Zheng L. McCleary C. MacK W. J. Chen Y. L. (2009). Longitudinal verbal fluency in normal aging, preclinical, and prevalent Alzheimer’s disease. Am. J. Alzheimers Dis. Other Demen. 24, 461–468. doi: 10.1177/1533317509345154 19759254 PMC2824246

[ref17] Connelly S. L. Hasher L. Zacks R. T. (1991). Age and reading: the impact of distraction. Psychol. Aging 6, 533–541. doi: 10.1037/0882-7974.6.4.533 1777141

[ref18] Craik F. I. M. Salthouse T. A. (2011). The Handbook of aging and cognition: third edition. New York: Psychology Press, 1–657.

[ref19] Crescentini C. Lunardelli A. Mussoni A. Zadini A. Shallice T. (2008). A left basal ganglia case of dynamic aphasia or impairment of extra-language cognitive processes? Neurocase 14, 184–203. doi: 10.1080/13554790802108380 18569743

[ref20] DeDe G. Flax J. K. (2016). “Language comprehension in aging” in Cognition, language and aging. ed. Wright H. H. (Amsterdam, The Netherlands: John Benjamins Publishing Company), 107–133.

[ref21] Doolen A. C. Radvansky G. A. (2021). A novel study: long-lasting event memory. Memory 29, 963–982. doi: 10.1080/09658211.2021.1953079 34278950

[ref22] Engelman M. Agree E. M. Meoni L. A. Klag M. J. (2010). Propositional density and cognitive function in later life: findings from the precursors study. J. Gerontol. Ser. B Psychol. Sci. Soc. Sci. 65, 706–711. doi: 10.1093/GERONB/GBQ064 20837676 PMC2954330

[ref23] Farias S. T. Chand V. Bonnici L. Baynes K. Harvey D. Mungas D.. (2012). Idea density measured in late life predicts subsequent cognitive trajectories: implications for the measurement of cognitive reserve. J. Gerontol. Ser. B Psychol. Sci. Soc. Sci. 67, 677–686. doi: 10.1093/GERONB/GBR162 22357642 PMC3478727

[ref24] Ferguson A. Spencer E. Craig H. Colyvas K. (2014). Propositional idea density in women’s written language over the lifespan: computerized analysis. Cortex 55, 107–121. doi: 10.1016/J.CORTEX.2013.05.012 23834740

[ref25] Fisher J. S. Radvansky G. A. (2018). Patterns of forgetting. J. Mem. Lang. 102, 130–141. doi: 10.1016/J.JML.2018.05.008

[ref26] Forbes-McKay K. Shanks M. F. Venneri A. (2013). Profiling spontaneous speech decline in Alzheimer’s disease: a longitudinal study. Acta Neuropsychiatrica 25, 320–327. doi: 10.1017/NEU.2013.16 25287871

[ref27] Fraser K. C. Meltzer J. A. Rudzicz F. (2016). Linguistic features identify Alzheimer’s disease in narrative speech. J. Alzheimers Dis. 49, 407–422. doi: 10.3233/JAD-150520 26484921

[ref28] Frederiksen C. H. Bracewell R. J. Breuleux A. Renaud A. (1990). “The cognitive representation and processing of discourse: function and dysfunction” in Discourse ability and brain damage: theoretical and empirical perspectives. eds. Joanette Y. Brownell H. (New York, NY: Springer Science & Business Media), 69–110.

[ref29] Fromm D. Forbes M. Holland A. Dalton S. G. Richardson J. MacWhinney B. (2017). Discourse characteristics in aphasia beyond the Western aphasia battery cutoff. Am. J. Speech Lang. Pathol. 26, 762–768. doi: 10.1044/2016_AJSLP-16-0071 28505222 PMC5829792

[ref30] Fromm D. Greenhouse J. Hou K. Russell G. A. Cai X. Forbes M.. (2016). Automated proposition density analysis for discourse in aphasia. J. Speech Lang. Hear. Res. 59, 1123–1132. doi: 10.1044/2016_JSLHR-L-15-0401 27657850 PMC5345557

[ref31] Gilchrist A. L. Cowan N. Naveh-Benjamin M. (2008). Working memory capacity for spoken sentences decreases with adult ageing: recall of fewer but not smaller chunks in older adults. Memory 16, 773–787. doi: 10.1080/09658210802261124 18671167 PMC2610466

[ref32] Glenberg A. M. Meyer M. Lindem K. (1987). Mental models contribute to foregrounding during text comprehension. J. Mem. Lang. 26, 69–83. doi: 10.1016/0749-596X(87)90063-5

[ref33] Harley T. A. (2016). The psychology of language: From data to theory. London, UK: Routledge. Available at: https://www.routledge.com/The-Psychology-of-Language-From-Data-to-Theory/Harley/p/book/9781848720893f

[ref34] Hoffman P. (2018). An individual differences approach to semantic cognition: divergent effects of age on representation, retrieval and selection. Sci. Rep. 8, 1–13. doi: 10.1038/s41598-018-26569-0 29802344 PMC5970266

[ref35] Hoffman P. Loginova E. Russell A. (2018). Poor coherence in older people’s speech is explained by impaired semantic and executive processes. Elife 7:e38907. doi: 10.7554/ELIFE.38907 30179156 PMC6150697

[ref36] Huber W. (1990). Text comprehension and production in aphasia: analysis in terms of micro-and macroprocessing. in Discourse Ability and Brain Damage. Eds. Joanette Y. Brownell H. H. Springer, New York, NY: Springer Series in Neuropsychology. doi: 10.1007/978-1-4612-3262-9_7

[ref37] Kemper S. J. (1993). “Language across the life span: coding manual” in Coding manual. ed. Lawrence K. S.. [*This may be an unpublished manuscript]

[ref38] Kemper S. Hoffman L. Schmalzried R. Herman R. Kieweg D. (2011). Tracking talking: dual task costs of planning and producing speech for young versus older adults. Aging Neuropsychol. Cognit. 18, 257–279. doi: 10.1080/13825585.2010.527317 PMC3091967 21140310

[ref39] Kemper S. Schmalzried R. Herman R. Leedahl S. Mohankumar D. (2009). The effects of aging and dual task demands on language production. Aging Neuropsychol. Cognit. 16, 241–259. doi: 10.1080/13825580802438868 PMC2674132 18982506

[ref40] Kemper S. Sumner A. (2001). The structure of verbal abilities in young and older adults. Psychol. Aging 16, 312–322. doi: 10.1037/0882-7974.16.2.312 11405318

[ref41] Kemper S. Thompson M. Marquis J. (2001). Longitudinal change in language production: effects of aging and dementia on grammatical complexity and propositional content. Psychol. Aging 16, 600–614. doi: 10.1037/0882-7974.16.4.600 11766915

[ref42] Kempler D. Van Lancker D. (2002). Effect of speech task on intelligibility in dysarthria: a case study of Parkinson’s disease. Brain Lang. 80, 449–464. doi: 10.1006/brln.2001.2602 11896652

[ref43] Kintsch W. (1998). Comprehension: a paradigm for cognition. Cambridge, UK: Cambridge University Press.

[ref44] Kintsch W. Keenan J. (1973). Reading rate and retention as a function of the number of propositions in the base structure of sentences. Cogn. Psychol. 5, 257–274. doi: 10.1016/0010-0285(73)90036-4

[ref45] Kintsch W. Monk D. (1972). Storage of complex information in memory: some implications of the speed with which inferences can be made. J. Exp. Psychol. 94, 25–32. doi: 10.1037/H0032781

[ref46] Kirshner H. S. (2012). Primary progressive aphasia and Alzheimer’s disease: brief history, recent evidence. Curr. Neurol. Neurosci. Rep. 12, 709–714. doi: 10.1007/S11910-012-0307-2 22932755

[ref47] Levelt W. J. M. (1993). Speaking: from intention to articulation. Cambridge, MA, USA: MIT Press.

[ref48] Levelt W. J. M. (2000). Producing spoken language: a blueprint of the speaker. The Neurocognition of Language, Eds. Brown Colin M. Hagoort Peter, Oxford University Press 82–122.

[ref49] Madden D. L. Sale M. V. Robinson G. A. (2019). Age-related differences in idea generation and selection for propositional language. Neuropsychol. Dev. Cogn. Sec. B Aging Neuropsychol. Cognit. 26, 486–506. doi: 10.1080/13825585.2018.1476668 29781396

[ref50] Mascali D. DiNuzzo M. Serra L. Mangia S. Maraviglia B. Bozzali M.. (2018). Disruption of semantic network in mild Alzheimer’s disease revealed by resting-state fMRI. Neuroscience 371:38. doi: 10.1016/J.NEUROSCIENCE.2017.11.030 29197559 PMC5809186

[ref51] Maseda A. Lodeiro-Fernández L. Lorenzo-López L. Núñez-Naveira L. Balo A. Millán-Calenti J. C. (2014). Verbal fluency, naming and verbal comprehension: three aspects of language as predictors of cognitive impairment. Aging Ment. Health 18, 1037–1045. doi: 10.1080/13607863.2014.908457 24797556

[ref52] Medina L. D. Rodriguez-Agudelo Y. Geschwind D. H. Gilbert P. E. Liang L. J. Cummings J. L.. (2011). Propositional density and apolipoprotein E genotype among persons at risk for familial Alzheimer’s disease. Dement. Geriatr. Cogn. Disord. 32, 188–192. doi: 10.1159/000333023 22134129 PMC3542946

[ref53] Mitzner T. L. Kemper S. (2003). Oral and written language in late adulthood: findings from the nun study. Exp. Aging Res. 29, 457–474. doi: 10.1080/03610730303698 12959878

[ref54] Mortensen L. Meyer A. S. Humphreys G. W. (2006). Age-related effects on speech production: a review. Lang. Cogn. Process 21, 238–290. doi: 10.1080/01690960444000278

[ref55] Mueller K. D. Koscik R. L. Turkstra L. S. Riedeman S. K. Larue A. Clark L. R.. (2016). Connected language in late middle-aged adults at risk for Alzheimer’s disease. J. Alzheimer’s Dis. 54, 1539–1550. doi: 10.3233/JAD-160252 27636838 PMC5137196

[ref56] Norman S. Kemper S. Kynette D. Cheung H. Anagnopoulos C. (1991). Syntactic complexity and adults’ running memory span. J. Gerontol. 46:P346-51. doi: 10.1093/GERONJ/46.6.P346 1940091

[ref57] Park D. C. Lautenschlager G. Hedden T. Davidson N. S. Smith A. D. Smith P. K. (2002). Models of visuospatial and verbal memory across the adult life span. Psychol. Aging 17, 299–320. doi: 10.1037/0882-7974.17.2.299 12061414

[ref58] Pushkar D. Basevitz P. Arbuckle T. Nohara-LeClair M. Lapidus S. Peled M. (2000). Social behavior and off-target verbosity in elderly people. Psychol. Aging 15, 361–374. doi: 10.1037/0882-7974.15.2.361 10879589

[ref59] Radvansky G. A. Dijkstra K. (2007). Aging and situation model processing. Psychon. Bull. Rev. 14, 1027–1042. doi: 10.3758/BF03193088 18229472

[ref60] Robinson G. A. (2013). Primary progressive dynamic aphasia and parkinsonism: generation, selection and sequencing deficits. Neuropsychologia 51, 2534–2547. doi: 10.1016/J.NEUROPSYCHOLOGIA.2013.09.038 24113151

[ref61] Robinson G. Blair J. Cipolotti L. (1998). Dynamic aphasia: an inability to select between competing verbal responses? Brain 121, 77–89. doi: 10.1093/BRAIN/121.1.77 9549489

[ref62] Robinson G. Shallice T. Bozzali M. Cipolotti L. (2010). Conceptual proposition selection and the LIFG: neuropsychological evidence from a focal frontal group. Neuropsychologia 48, 1652–1663. doi: 10.1016/J.NEUROPSYCHOLOGIA.2010.02.010 20153763

[ref63] Robinson G. Shallice T. Cipolotti L. (2006). Dynamic aphasia in progressive supranuclear palsy: a deficit in generating a fluent sequence of novel thought. Neuropsychologia 44, 1344–1360. doi: 10.1016/J.NEUROPSYCHOLOGIA.2006.01.002 16504225

[ref64] Sachs J. S. (1967). Recopition memory for syntactic and semantic aspects of connected discourse. Percept. Psychophys. 2, 437–442. doi: 10.3758/BF03208784

[ref65] Salis C. (2011). Understanding of auditory discourse in older adults: the effects of syntax and working memory. Aphasiology 25, 529–539. doi: 10.1080/02687038.2010.527998

[ref66] Salthouse T. A. (1990). Working memory as a processing resource in cognitive aging. Dev. Rev. 10, 101–124. doi: 10.1016/0273-2297(90)90006-P

[ref67] Salthouse T. A. (1996). The processing-speed theory of adult age differences in cognition. Psychol. Rev. 103, 403–428. doi: 10.1037/0033-295X.103.3.403 8759042

[ref68] Sand Aronsson F. Kuhlmann M. Jelic V. Östberg P. (2021). Is cognitive impairment associated with reduced syntactic complexity in writing? Evidence from automated text analysis. Aphasiology 35, 900–913. doi: 10.1080/02687038.2020.1742282

[ref69] Smolík F. Stepankova H. Vyhnálek M. Nikolai T. Horáková K. Matějka S. (2016). Propositional density in spoken and written language of Czech-speaking patients with mild cognitive impairment. J. Speech Lang. Hear. Res. 59, 1461–1470. doi: 10.1044/2016_JSLHR-L-15-0301 27960195

[ref70] Snowdon D. A. Greiner L. H. Markesbery W. R. (2000). Linguistic ability in early life and the neuropathology of Alzheimer’s disease and cerebrovascular disease: findings from the nun study. Ann. N. Y. Acad. Sci. 903, 34–38. doi: 10.1111/J.1749-6632.2000.TB06347.X 10818486

[ref71] Snowdon D. A. Kemper S. J. Mortimer J. A. Greiner L. H. Wekstein D. R. Markesbery W. R. (1996). Linguistic ability in early life and cognitive function and Alzheimer’s disease in late life: findings from the nun study. JAMA 275, 528–532. doi: 10.1001/JAMA.275.7.528 8606473

[ref72] Soares F. C. de Oliveira T. C. G. de Macedo L. D. E. D. Tomás A. M. Picanço-Diniz D. L. W. Bento-Torres J.. (2014). CANTAB object recognition and language tests to detect aging cognitive decline: an exploratory comparative study. Clin. Interv. Aging 10, 37–48. doi: 10.2147/CIA.S68186 25565785 PMC4279672

[ref73] Spencer E. Craig H. Ferguson A. Colyvas K. (2012). Language and ageing - exploring propositional density in written language - stability over time. Clin. Linguist. Phonet. 26, 743–754. doi: 10.3109/02699206.2012.673046 22876766

[ref74] Stine E. A. L. Hindman J. (1994). Age differences in reading time allocation for propositionally dense sentences. Aging Neuropsychol. Cognit. 1, 2–16. doi: 10.1080/09289919408251446

[ref75] Stine-Morrow E. A. L. Miller L. M. S. Hertzog C. (2006). Aging and self-regulated language processing. Psychol. Bull. 132, 582–606. doi: 10.1037/0033-2909.132.4.582 16822168 PMC2246038

[ref76] Turner A. Greene E. (1977). The construction and use of a propositional text base. Technical Report No. 63. Boulder: Institute for the Study of Intellectual Behavior, University of Colorado.

[ref77] Van Dijk T. A. Kintsch W. (1983). Strategies of discourse comprehension. Available at: https://www.academia.edu/download/77815914/Teun_20A_20van_20Dijk_20__20Walter_20Kintsch_20-_20Strategies_20of_20Discourse_20Comprehension.pdf

[ref9001] Véliz M. Hernández M. Sáez K. Riffo B. Sáez Y. (2013). Sentence production by elder and young adults under a controlled situation[Oraciones producidas por adultos mayores y adultos jóvenes en una situación controlada]. Onomazein 27, 241–257. doi: 10.7764/onomazein.27.16

[ref78] Venneri A. Jahn-Carta C. De Marco M. Quaranta D. Marra C. (2018). Diagnostic and prognostic role of semantic processing in preclinical Alzheimer’s disease. Biomark. Med 12, 637–651. doi: 10.2217/BMM-2017-0324 29896968

[ref79] Venneri A. Mitolo M. De Marco M. (2016). Paradigm shift: semantic memory decline as a biomarker of preclinical Alzheimer’s disease. Biomark. Med 10, 5–8. doi: 10.2217/BMM.15.53 26642376

[ref80] Webster J. Morris J. Howard D. Garraffa M. (2018). Reading for meaning: what influences paragraph understanding in aphasia? Am. J. Speech Lang. Pathol. 27, 423–437. doi: 10.1044/2017_AJSLP-16-0213 29497753

[ref81] Williams J. M. G. Barnhofer T. Crane C. Hermans D. Raes F. Watkins E.. (2007). Autobiographical memory specificity and emotional disorder. Psychol. Bull. 133, 122–148. doi: 10.1037/0033-2909.133.1.122 17201573 PMC2834574

[ref82] Wingfield A. Stine-Morrow E. A. L. (2000). “Language and speech” in The handbook of aging and cognition. eds. Craik F. I. M. Salthouse T. A.. 2nd ed (Mahwah, NJ, USA: Lawrence Erlbaum Associates Publishers), 359–416.

[ref83] Zahn R. Juengling F. Bubrowski P. Jost E. Dykierek P. Talazko J.. (2004). Hemispheric asymmetries of hypometabolism associated with semantic memory impairment in Alzheimer’s disease: a study using positron emission tomography with fluorodeoxyglucose-F18. Psychiatry Res. 132, 159–172. doi: 10.1016/J.PSCYCHRESNS.2004.07.006 15598550

